# Genes and pathways associated with pregnancy loss in dairy cattle

**DOI:** 10.1038/s41598-021-92525-0

**Published:** 2021-06-25

**Authors:** Anil Sigdel, Rafael S. Bisinotto, Francisco Peñagaricano

**Affiliations:** 1 Department of Animal and Dairy Sciences, University of Wisconsin-Madison, Madison, WI 53706 USA; 2 Department of Large Animal Clinical Sciences, University of Florida, Gainesville, FL 32611 USA

**Keywords:** Genetics, Animal breeding, Genetic association study, Genomics, Quantitative trait

## Abstract

Pregnancy loss directly impairs reproductive performance in dairy cattle. Here, we investigated genetic factors associated with pregnancy loss following detection of a viable embryo around 42 days of gestation. The objectives of this study were to perform whole-genome scans and subsequent gene-set analyses for identifying candidate genes, functional gene-sets and gene signaling pathways implicated in pregnancy loss in US Holstein cows. Data consisted of about 58,000 pregnancy/abortion records distributed over nulliparous, primiparous, and multiparous cows. Threshold models were used to assess the binary response of pregnancy loss. Whole‐genome scans identified at least seven genomic regions on BTA2, BTA10, BTA14, BTA16, BTA21, BTA24 and BTA29 associated with pregnancy loss in heifers and lactating cows. These regions harbor several candidate genes that are directly implicated in pregnancy maintenance and fetal growth, such as *CHST14, IGF1R, IGF2, PSEN2, SLC2A5* and *WNT4*. Moreover, the enrichment analysis revealed at least seven significantly enriched processes, containing genes associated with pregnancy loss, including calcium signaling, cell–cell attachment, cellular proliferation, fetal development, immunity, membrane permeability, and steroid metabolism. Additionally, the pathway analysis revealed a number of significant gene signaling pathways that regulate placental development and fetal growth, including Wnt, Hedgehog, Notch, MAPK, Hippo, mTOR and TGFβ pathways. Overall, our findings contribute to a better understanding of the genetic and biological basis of pregnancy loss in dairy cattle and points out novel strategies for improving pregnancy maintenance via marker‐assisted breeding.

## Introduction

Reproductive performance is a major determinant of efficient dairy production. Given its importance, there has been a considerable effort in improving dairy cow reproductive efficiency through nutrition, cow comfort, control of ovulation, and, more recently, inclusion of reproductive traits into breeding objectives and selection programs^1^. Yet, reproductive performance remains suboptimal in many dairy herds. Pregnancy loss is arguably the major factor contributing to poor reproductive performance in dairy cattle. In fact, fertilization is observed in 70% to 75% of high producing dairy cows subjected to insemination whereas embryonic and fetal losses result in calving rates that range between 30 and 35%^2,3^. Pregnancy losses during the embryonic period (i.e., first 42 days of gestation) have been estimated around 25% to 40% whereas pregnancy losses during the fetal period (i.e., after 42 days of gestation) range between 2 and 12%^4^. The cost of a pregnancy loss in dairy cattle ranges from US$90 to $1900 depending on the gestational stage at which pregnancy is lost and is associated with longer calving interval, reduced availability of potential replacement heifers, decreased milk production, increased insemination, veterinary, and labor costs, and premature culling^5,6^. Although pregnancy losses during the embryonic period are more frequent, pregnancy losses during the fetal period have a much greater economic impact due to extended calving intervals and early culling of productive cows^7^. Fetal loss may also result in the retention of fetal membranes and the development of endometritis, which further reduces reproductive performance and increases veterinary and labor costs. Therefore, pregnancy loss during the fetal period should not be overlooked in breeding schemes aimed at improving the reproductive performance of dairy cattle.

Numerous factors may cause fetal loss, including infectious diseases, toxic agents, heat stress, and genetic factors. Indeed, previous studies have reported that there is a substantial genetic variation underlying fetal loss in dairy catlle^8–10^. Known genetic factors include lethal recessive alleles, such as complex vertebral malformation (CVM) and deficiency of uridine monophosphate synthase (DUMPS), and an increasing list of recessive haplotypes^11^. Recently, Gershoni and colleagues identified *ABCA9* gene as a candidate gene for early abortions in Israeli dairy cattle^10^. Interestingly, *ABCA9* gene is located within an ATP-binding cassette (ABC) genes cluster on BTA19, and the ABC family is the major class of primary active transporters in the placenta. Overall, current evidence suggests that fetal loss is influenced by genetic factors, and hence it could be improved by genetic means.

The main objective of this study was to characterize the genetic basis of pregnancy loss in US Holstein cattle. We investigated pregnancy loss following the detection of a viable embryo, and hence, we analyzed direct records of pregnancy loss, rather than indirect indicators of pregnancy loss, such as long insemination intervals. We performed whole-genome scans to identify candidate genes associated with pregnancy loss. Given that association studies detect, in general, only the most significant genetic markers, we also applied SNP-based gene-set enrichment tools to gain additional insight into the biological processes and molecular mechanisms that could be affecting pregnancy loss. Our findings will contribute to a better understanding of the genetic variants and complex biological mechanisms underlying pregnancy loss in dairy cattle, in addition to providing novel tools for improving reproductive efficiency via marker-assisted selection.

## Materials and methods

### Phenotypic and genotypic data

Records from nulliparous (heifers), primiparous (first lactation cows) and multiparous (second lactation cows) Holsteins diagnosed as pregnant 39 ± 3 days after breeding were compiled from a single dairy herd located in Florida, USA between 2001 and 2019 (Table 1). Records of pregnancy loss were obtained from veterinary pregnancy examinations. Females were assumed to undergo pregnancy loss if a negative veterinary examination was reported in subsequent pregnancy checks, after the initial confirmation of pregnancy at 42 days. Pregnancy loss was recorded as a binary trait, i.e., Y = 1 if the heifer or cow diagnosed as pregnant 39 ± 3 days after breeding was diagnosed as non-pregnant in subsequent examinations, and Y = 0 if the heifer or cow remained pregnant in subsequent examinations. The pedigree file was created by tracing the pedigree of cows back five generations.

**Table 1:** Summary of data by parity.

Data	Nulliparous	Primiparous	Multiparous
No. records	23,910	20,495	13,793
Pregnancy loss, %	8.3	13.5	13.7
No. animals in pedigree	33,149	29,209	20,883
No. genotyped animals	11,847	8864	4522
No. genotyped sires	1097	1012	822
No. genotyped dams	10,750	7852	3700

Genotype data for 72,444 SNPs across the bovine genome were available for heifers and cows with records, and also sires in the pedigree. The SNP information was updated to the new bovine reference genome ARS-UCD 1.2. Quality control was conducted both at the level of animal and SNP markers. Animals were removed from the analysis if they had a call rate < 95%. The SNPs were removed if they mapped to the sex chromosomes, had a call rate < 95%, minor allele frequency < 1%, or if they failed the Hardy–Weinberg equilibrium test. After quality control, a total of 69,051 SNP markers were retained for subsequent genomic analyses. Summary statistics of the final dataset used for the analyses are presented in Table 1.

### Statistical model

The threshold model, also known as probit model, was used to evaluate the incidences of pregnancy loss in each of the three parities, i.e., nulliparous, primiparous and multiparous. This model describes the variation observed at a binary response variable (*Y*, either 0 or 1) using an underlying random variable $\mathbf{z}$, known as liability,

$$\mathbf{z}=\boldsymbol{\upeta }+\boldsymbol{\upepsilon }$$

where $\boldsymbol{\upeta }$ is a vector of linear predictors and $\boldsymbol{\upepsilon }$ is a vector of independent and identically distributed standard normal random variables, **ε** ~ $N(0,1)$. Here, pregnancy loss is $Y=1$ if the underlying liability $(z)$ is greater than zero, i.e., $\mathbf{Y}={1 \; \text{if} \quad \mathbf{z}>\mathbf{0}}$; **0** otherwise. Therefore, the conditional probability of observing a pregnancy loss event is P $\left(\mathbf{Y}=\mathbf{1}|\boldsymbol{\upeta }\right)$ = ${\Phi }$($\boldsymbol{\upeta }$) where ${\Phi }$(.) is the standard normal cumulative distribution. The likelihood function for the binary response variable then becomes:

$$p\left(\mathbf{Y}|\boldsymbol{\upeta }\right)=\prod {\Phi }{\left(\boldsymbol{\upeta }\right)}^{\mathbf{Y}}\cdot {\left[1-{\Phi }\left(\boldsymbol{\upeta }\right)\right]}^{\mathbf{1}-\mathbf{Y}}$$

where $\mathbf{Y}$ and $\boldsymbol{\upeta }$ are the vectors of the binary responses and the linear predictors, respectively.

For pregnancy loss incidence, the linear predictor $\boldsymbol{\upeta }$ has the following form:

$$\boldsymbol{\upeta }=\mathbf{X}\boldsymbol{\upbeta }+{\mathbf{Z}}_{1}\mathbf{a}+{\mathbf{Z}}_{2}\mathbf{s}\mathbf{s}$$

where $\boldsymbol{\upbeta }$ is a vector for fixed effects of year-season of breeding (39 levels), type of breeding (2 levels; insemination or embryo transfer), days in milk (DIM) at breeding (3 levels; only for lactating cows), and occurrence of uterine diseases (binary trait, only for lactating cows), $\mathbf{a}$ is a vector of random additive genetic effects, and $\mathbf{s}\mathbf{s}$ is a vector of random service sire effects. The matrices $\mathbf{X}$, ${\mathbf{Z}}_{1}$, and ${\mathbf{Z}}_{2}$ are the incidence matrices relating phenotypic records to fixed, animal, and sire effects, respectively. Random effects were assumed to follow a multivariate normal distribution,

$$\left( {\left. {\begin{array}{{l}} \mathbf{a}\\ \mathbf{ss} \end{array}} \right|\sigma _a^2,\sigma _{ss}^2} \right) \sim N\left[ {\mathbf{0},\left( {\begin{array}{{ll}} {\mathbf{H}\sigma _a^2}& \quad 0\\ 0& \quad {\mathbf{I}\sigma _{ss}^2} \end{array}} \right)} \right]$$

where $\mathbf{a}$ and $\mathbf{ss}$ are the vectors of animal and service sire effects respectively; ${\sigma}_{a}^{2}$ and ${\sigma}_{ss}^{2}$ are animal and service sire variances, respectively. Here, the classical pedigree relationship matrix $\mathbf{A}$ is replaced by $\mathbf{H}$ which combines pedigree and genotypic information. This method is known as single-step genomic best linear unbiased prediction (ssGBLUP). The matrix ${\mathbf{H}}^{-1}$ was calculated as follows,

$${{\mathbf{H}}^{-1}=\mathbf{A}}^{-1}+\left[\begin{array}{ll}0& 0\\ 0& {\mathbf{G}}^{-1}-{\mathbf{A}}_{22}^{-1}\end{array}\right]$$

where ${\mathbf{G}}^{-1}$ is the inverse of the genomic relationship matrix and ${\mathbf{A}}_{22}^{-1}$ is the inverse of the pedigree-based relationship matrix for genotyped animals. Here, **G** matrix was obtained using the observed allele frequency of the genotypes. The **G** was created considering heifers and cows with both genotypes and abortion records, plus genotyped sires in the pedigree. The pedigree relationship **A** matrix was created using a five-generation pedigree file obtained from the Council of Dairy Cattle Breeding (Table 1).

### Model implementation

Threshold models were implemented in a Bayesian framework using THRGIBBS1F90 (version 1.93)^12^. A Monte Carlo Markov chain approach through Gibbs sampling was used to obtain features of the posterior distribution. Inferences were based on 500,000 samples obtained after discarding the first 100,000 samples as burn in. A thinning interval of 100 was used to compute statistics of the posterior distribution. Convergence diagnostics of Markov chain Monte Carlo sampling output were carried out by visual inspection of trace plots of key parameters such as variance components.

### Genome-wide association mapping

Candidate genomic regions associated with pregnancy loss in each parity under study were identified based on the amount of genetic variance explained by 2.0 Mb moving windows of adjacent SNPs. The SNP effects were estimated as $\widehat{\boldsymbol{s}}$** =** $\boldsymbol{D}\boldsymbol{M}\boldsymbol{^{\prime}}\left[\boldsymbol{M}\boldsymbol{D}{\left.\boldsymbol{M}\boldsymbol {^{\prime}} \right]}^{-1}\right.{\widehat{\boldsymbol{a}}}_{\boldsymbol{g}}$, where $\widehat{\boldsymbol{s}}$ is the vector of SNP marker effects, $\boldsymbol{D}$ is a diagonal matrix of weights of SNPs, $\boldsymbol{M}$ is a matrix relating genotype of each SNP marker to observations and ${\widehat{\boldsymbol{a}}}_{\boldsymbol{g}}$ is the vector of GEBVs for genotyped individuals^13^. The percentage of genetic variance explained by a given 2.0 Mb region was then calculated as

$$\frac{{{\mathop{\text var}} \left( {{u_i}} \right)}}{{\sigma _a^2}} \times 100 = \frac{{{\mathop{\text var}} \left( {\sum\nolimits_{j = 1}^B {{M_j}{S_j}} } \right)}}{{\sigma _a^2}}$$

where ${u}_{i}$ is the genetic value of the ${i}$th genomic region under consideration, *B* is the total number of adjacent SNPs within 2.0 Mb region, ${M}_{j}$ is the genotype code of ${j}$th marker, ${S}_{j}$  is the marker effect of the ${j}$th SNP within the ${i}$th region. In this study, all SNPs were equally weighted. All these ssGBLUP calculations were performed using POSTGSF90 (version 3.08) of BLUPF90 family of programs^14^.

### Gene-set enrichment analysis

#### Mapping SNPs to genes

The first step in gene-set enrichment analysis is to map SNPs to genes. The Bioconductor *R* package biomaRt^15^ and the latest bovine reference genome ARS-UCD 1.2. were used to map SNPs to genes. Specifically, SNPs were assigned to genes if they were located within the genomic sequence of an annotated gene or within 15 kb either upstream or downstream the gene. The distance of 15 kb was used to capture proximal regulatory regions and other functional sites that may lie outside (e.g., promoter region) but close to each gene. If a SNP was found to be located within or close to more than one gene, all these genes were included in subsequent analyses. A 5% threshold of the SNP effects was used to define relevant SNPs; hence, a gene was associated with pregnancy loss if it contained at least one SNP whose effect was in the top 5% of the distribution.

#### Assignment of genes to functional categories

Different databases including GO, MeSH, Reactome, InterPro and MsigDB were used to define functional sets of genes. Genes assigned to the same functional category can be regarded as more closely related in terms of biology or function than random sets of genes. Only gene-sets, biological processes, molecular mechanisms, or gene signaling pathways with 10 or more genes were considered in these analyses.

#### Association analysis between functional categories and pregnancy loss

The significant association of a given functional term with pregnancy loss was analyzed using the Fisher’s exact test, a test of proportions based on the cumulative hypergeometric distribution^16^. This test was performed to search for an overrepresentation of relevant genes in each functional category. The *P*-value of observing $g$ relevant genes in the term was calculated by

$$Pvalue = 1 - \frac{{\sum\nolimits_{i = 0}^{g - 1} {\left( {\begin{array}{l} S\\ i \end{array}} \right)\left( {\begin{array}{l} {N - 1}\\ {K - 1} \end{array}} \right)} }}{{\left( {\begin{array}{l} N\\ K \end{array}} \right)}}$$

where $S$ is the total number of relevant genes associated with pregnancy, $N$ is the total number of genes that were analyzed, and $K$ is the total number of genes in the term considered. The gene-set enrichment analysis was performed using the *R* package EnrichKit, developed by Lihe Liu and Francisco Peñagaricano, available at https://github.com/liulihe954/EnrichKit.

## Results and discussion

Improving reproductive efficiency is one of the most important goals for the dairy industry worldwide. Yet, reproductive efficiency remains suboptimal in most of the dairy herds. Pregnancy loss is recognized as one of the most important factors that reduces reproductive performance and dairy farm profitability. In fact, pregnancy loss is expensive, and the cost increases rapidly as the gestation progresses. Most genetic studies have focused on early pregnancy loss during the embryonic period, whereas late pregnancy loss during the fetal period has been largely overlooked. Despite having a smaller incidence, late fetal losses have a much greater economic impact compared with earlier embryonic losses^5,6^. As such, this study was conducted to unravel individual genes, functional gene-sets, biological processes, and gene signaling pathways underlying pregnancy losses during the fetal stage in US Holstein cattle.

### Gene mapping

**Figure 1:**
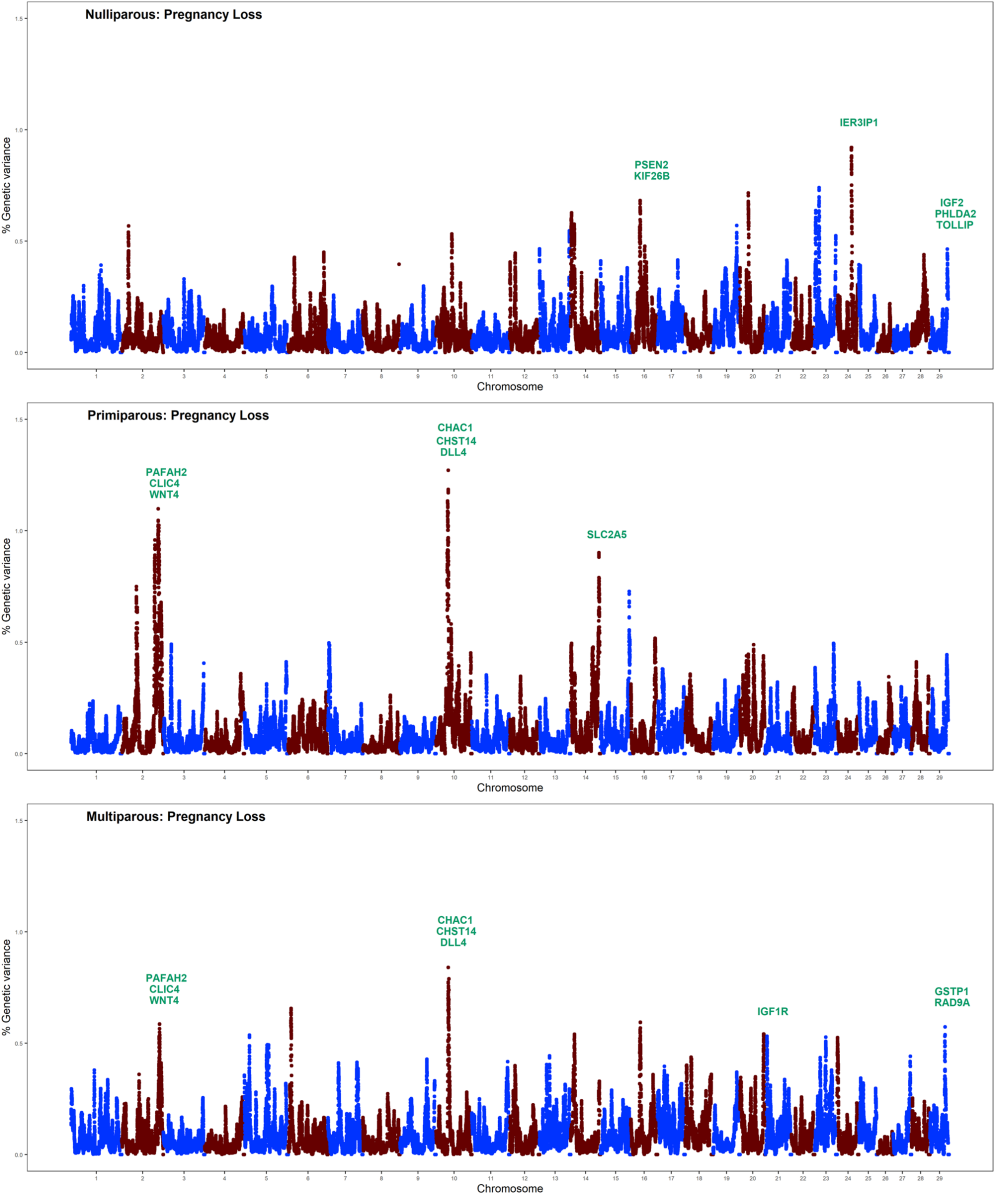
Whole-genome scans for pregnancy loss across the first three parities in Holstein dairy cows: Percentage of additive genetic variance explained by 2.0 Mb single nucleotide polymorphism (SNP)-windows across the genome. Genes directly implicated in pregnancy maintenance and fetal growth are highlighted in green.

Single-step genomic BLUP methodology was utilized to identify genomic regions and putative candidate genes affecting pregnancy loss in nulliparous, primiparous and multiparous cows. This method combines all available phenotypic, pedigree, and genotypic data and fits all SNPs simultaneously. Figure 1 displays Manhattan plots for pregnancy loss across the three parities. Genomic regions and candidate genes were identified based on the amount of genetic variance explained by 2.0 Mb SNP-windows across the entire bovine genome. Note that most of the genomic regions associated with pregnancy loss are parity specific. In other words, there is a remarkable genotype-by-parity interaction, regions and major genes associated with pregnancy loss vary across lactations. The low genetic correlations (calculated as correlations between SNP effects) across parities support these findings (Table 2).

**Table 2:** Genetic parameters for pregnancy loss in Holstein cattle: heritability estimates in the diagonal and genetic correlations (calculated as correlations between SNP effects) above the diagonal.

	Nulliparous	Primiparous	Multiparous
Nulliparous	0.015 [0.009–0.042]	0.521	0.011
Primiparous		0.084 [0.033–0.169]	0.216
Multiparous			0.182 [0.130–0.365]

Genomic scan identified peaks on BTA16, BTA24, and BTA29 associated with pregnancy loss in nulliparous heifers. Interestingly, these genomic regions harbor putative candidate genes, such as *PSEN2* and *KIF26B* (BTA16, 29.68–31.66 Mb), *IER3IP1* (BTA24, 45.18–47.18 Mb), and *IGF2, PHLDA2*, and *TOLLIP* (BTA29, 48.33–50.31 Mb). The *PSEN2* gene is associated with notch signaling pathway and is involved in cellular proliferation, differentiation and placental angiogenesis^17^. Knock-out of *PSEN2* in mice exhibits abnormal tissue development, including defective somite formation leading to intrauterine growth restrictions and fetal death^18^. The *KIF26B* gene of the kinesin family is involved in fetal kidney development and many crucial cellular processes, including cell proliferation, differentiation, migration^19^. The *IER3IP1* gene is highly expressed in the fetal brain cortex and regulates fetal brain development^20^. The *IGF2* gene is involved in fetal growth as it controls the supply of oxygen nutrients available to the fetus through placental circulation^21^. The *IGF2* gene affects the metabolism, mitogenesis and differentiation of many cells including the placenta, while mutations in this gene cause fetal death by altering the supply of oxygen and nutrients^22^. Maternal expression of the *PHLDA2* gene has been linked to fetal growth restrictions resulting in poor perinatal outcomes such as fetal distress and fetal death thereby supporting a role of the *PHLDA2* gene in fetal growth and placental development^23^. In mice, maternal expression of the *PHLDA2* gene results in the termination of pregnancy^24^. The *TOLLIP* gene is a Wnt signaling associated gene involved in the immune regulation at the maternal–fetal interface. Activation of *TOLLIP* gene at the maternal–fetal interface produces Tumor necrosis factor-α (TNF-α) that stimulates apoptosis of villi-trophoblast cells, which suggests that *TOLLIP* gene may be a potential risk factor for pregnancy failure^25^. Our results confirm a genetic basis of pregnancy loss in nulliparous heifers and mapping of potential candidate genes at associated loci identified several genes (*PSEN2, KIF26B*, *IER3IP1, IGF2, PHLDA2*, and *TOLLIP*) with a plausible biological role in placental biology, fetal growth, and immune modulation.

Two genomic regions on BTA2 and BTA10 were strongly associated with pregnancy loss in both primiparous and multiparous cows. These genomic regions harbor strong candidate genes implicated in cellular functions related to placental development and fetal growth, such as *PAFAH2, CLIC4,* and *WNT4* (BTA2, 126.70–128.26 Mb), and *CHAC1, CHST14*, and *DLL4* (BTA10, 33.62–35.56 Mb). The *PAFAH2* gene has been implicated in various stages of reproduction, including implantation, fetal development, and parturition^26^. The *PAFAH2* gene stimulates the formation of IP3 and DAG and increases intracellular calcium^27^. Calcium is involved in bone formation and fetal mineralization. The *CLIC4* gene is implicated in mid gestational brain differentiation and neurogenesis^28^. The *WNT4* gene, a member of the Wnt signaling pathway, is implicated in the development of organ systems and extra embryonic tissues, particularly vascularization of the placenta. It promotes placental development through trophoblast lineage determination, chorioallantois fusion, and placental branching morphogenesis^29^. Failure in proper placental development creates an anaerobic environment and causes oxidative stress and fetal death^30^. The *CHAC1* gene is an endoplasmic reticulum stress gene that is involved in apoptosis initiation and execution^31^. The *CHST14* gene is implicated in dermatan sulfate biosynthetic process. In mice, knock-out of *CHST*14 gene produces changes in the placenta, including reduced weight, alterations in the vascular structure, and ischemic and/or necrotic-like changes, indicating this gene is essential for placental vascular development and perinatal survival of the fetus^32^. In brief, whole-genome scans revealed two common genomic regions on BTA2 and BTA10 as associated with pregnancy loss in primiparous and multiparous cows, suggesting that genes important for pregnancy loss are common in lactating cows. These genomic regions harbor candidate genes important for fetal development, calcium regulation, fetal-maternal circulation, and immunity.

For primiparous cows, one genomic region located on BTA14 explained almost 1.0% of additive genetic variance for pregnancy loss. This genomic region harbors several genes, including *SLC2A5* (BTA14, 81.94–83.93 Mb). The *SLC2A5* gene, a solute carrier transporter, is expressed in uterine-placental interface during mid to late pregnancy stage. The *SLC2A5* gene transports fructose from placenta to fetus and hence, supports the growth and development of fetus^33^. For multiparous cows, two different genomic regions on BTA21 (72.83–92.67 Mb) and BTA29 (43.42–45.42 Mb) explained more than 0.5% of the additive genetic variance for pregnancy loss. The genomic region on BTA21 harbors several genes including *IGF1R* which is involved in fetal growth and development. The *IGF1R* gene is implicated in the transfer of nutrients to the fetus and promotes anabolic events during the fetal stage of development^34^. The *RAD9A* gene, a key gene of the DNA damage response pathway, is involved in genomic stability and embryo-fetal development; deletion of mouse *RAD9A* gene results in post-zygotic loss and reduced fertility^35^. The *GSTP1* gene on BTA29 is important for detoxification of toxic compounds that pass the placental barrier and protects the placenta and fetus from toxic products^36^.

Overall, our whole-genome scans have detected several genomic regions and candidate genes associated with pregnancy loss in both non-lactating heifers and lactating primiparous and multiparous cows. Interestingly, these genomic regions harbor candidate genes that have diverse biological roles in placental development, fetal growth, pregnancy maintenance, immune modulation, calcium signaling, vascularization and organogenesis. These genomic regions are excellent candidates for future research to identify functional mutations associated with pregnancy loss in dairy cattle.

### Gene-set analysis

Whole-genome association mapping evaluated 72,444 SNP markers, however, only SNPs located within annotated genes or at most 15 kb upstream or downstream from annotated genes were used for the gene-set analysis. This set of SNPs defined a total of 20,087 genes in the new ARS-UCD 1.2. bovine reference genome. A subset of 1903 genes in nulliparous heifers, 1892 genes in primiparous, and 1882 genes in multiparous cows were considered as significantly associated with pregnancy loss, i.e., set of genes flagged by at least one SNP located in the top 5% of the SNP effect distribution.

**Figure 2:**
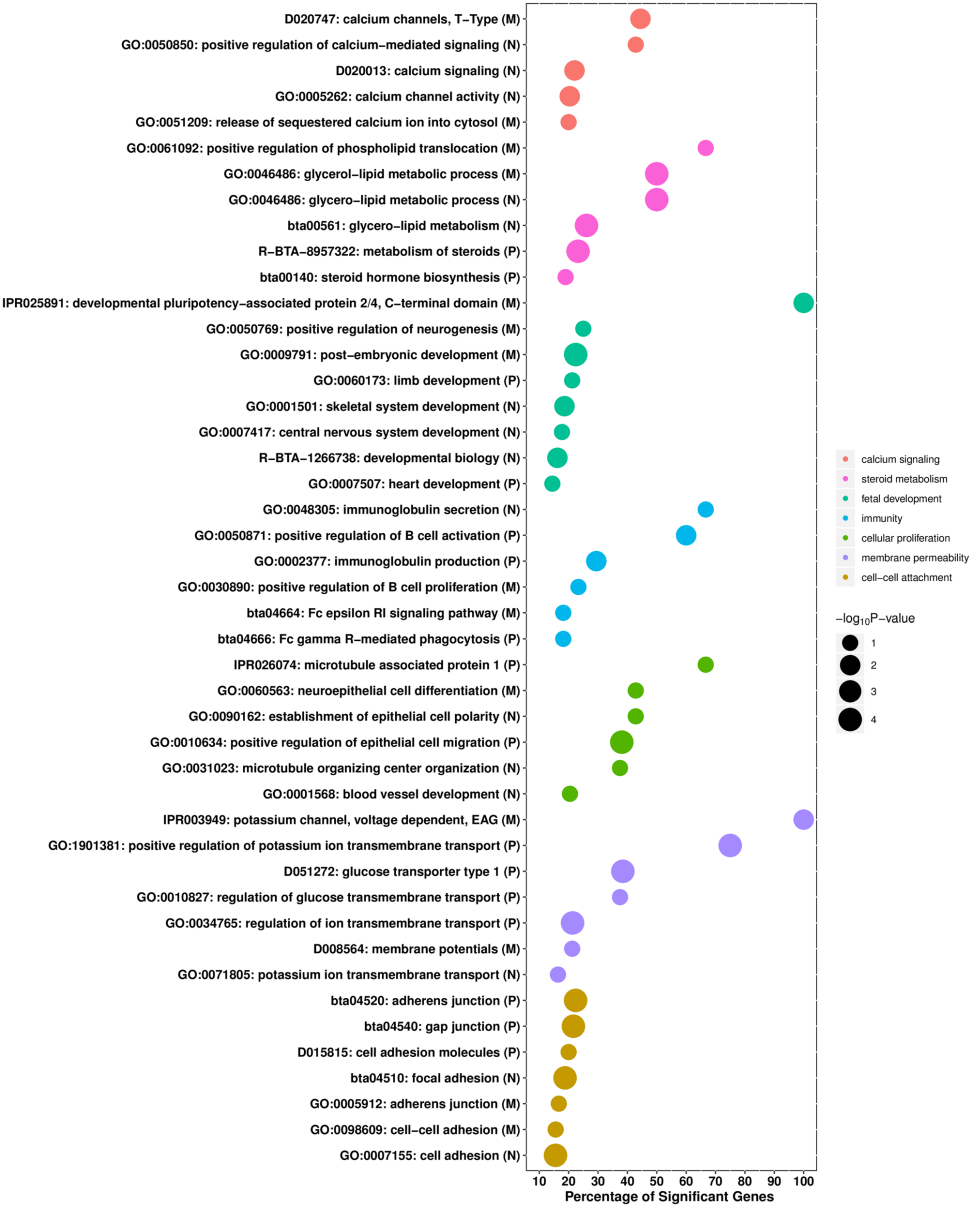
Functional terms and pathways significantly enriched with genes associated with pregnancy loss. Several annotation databases were analyzed, including GO, Medical Subject Headings, InterPro, Reactome and MSigDB. The y-axis displays the names of gene-sets associated with pregnancy loss, and letters within parenthesis represent the three parities (N: Nulliparous; P: Primiparous and M: Multiparous). The size of the dots represents the significance of enrichment (− log_10_ *P-*Value, Fisher’s exact test) and x-axis represents the percentage of significant genes in each functional term.

Figure 2 shows the most relevant biological terms and pathways associated with pregnancy loss. Note that our gene-set analysis used different public annotation databases, including GO, MeSH, InterPro, Reactome, and MSigDB. Across these annotation databases, genome-wide association signals for pregnancy loss were highly enriched in at least seven groups of gene-sets, namely calcium signaling, steroid metabolism, fetal development, immunity, cellular proliferation, membrane permeability and cell–cell attachment. Supplementary Tables S1–3 report the full list of significant biological terms, including term ID, term name, total number of genes, number of significant genes and Fisher’s *P*‐value.

Noticeably, some of the enriched terms are directly involved in fetal growth and organogenesis, such as *post-embryonic development* (GO:0009791), *limb development* (GO:0060173), *skeletal system development* (GO:0001501), *central nervous system development* (GO:0007417), *positive regulation of neurogenesis* (GO:0050769), *heart development* (GO:0007507), *developmental biology* (R-BTA-1266738), and *developmental pluripotency-associated protein 2/4, C-terminal domain* (R025891). These significant terms contain relevant genes, such as *BMPR1B, BMPR1, MAP2K1,* and *DLG4*, all of which are involved in embryogenesis, development of multiple organs systems, and maintenance of tissues homeostasis^37–39^.

The significant terms enriched in genes involved in calcium signaling include *positive regulation of calcium-mediated signaling* (GO:0050850), *calcium channel activity* (GO:0005262), *release of sequestered calcium ion into cytosol* (GO:0051209), *calcium channels, T-type* (D020747) and *calcium signaling* (D020013). The calcium ion is an important secondary messenger involved in proper placental development and function, critical for the growth and survival of the fetus. These calcium‐related gene‐sets contained several significant genes, namely *ITPR2, ITPR3,* and *PLD1*, which are associated with cell proliferation, survival, differentiation, cytoskeletal organization, and maintenance of the fetal-maternal connections and viability of the fetus^40,41^.

Steroid metabolism was identified as a biological process significantly enriched with genes implicated in pregnancy maintenance. Functional terms or pathways associated with steroid metabolism include *positive regulation of phospholipid translocation* (GO:0061092), *glycerol-lipid metabolic process* (GO:0046486), *glycero-lipid metabolism* (bta00561), *metabolism of steroids* (R-BTA-8957322) and *steroid hormone biosynthesis* (bta00140). These terms were enriched with relevant genes, including *DGKG, DGAT2*, *GPAT3,* and *ATP8A1*, which maintain the steroid hormones, namely progesterone, estrogens, gluco- and mineralocorticosteroids between the mother, fetus, and placenta. Steroid hormones are involved in several events during pregnancy, including proliferation of trophoblasts and healthy progression of pregnancy^42,43^.

There were also some relevant functional terms related to immunity, including *immunoglobulin secretion* (GO:0048305), *positive regulation of B cell activation* (GO:0050871), *immunoglobulin production* (GO:0002377), *positive regulation of B cell proliferation* (GO:0030890) and *Fc epsilon RI signaling pathway* (bta04664) and *Fc gamma R-mediated phagocytosis* (bta04666). These functional gene-sets harbor many relevant genes, such as *CD81, CD40, CLCF1,* and *MAPK12*, which establish immune tolerance at the fetal-maternal interface and allow allogeneic fetal trophoblasts to invade maternal tissues, which in turn permits fetal and placental development^44,45^.

Cell–cell attachment and cellular proliferation were identified as two important biological processes significantly enriched with genes implicated in pregnancy loss. Functional terms associated with cell–cell attachment include *adherens junction* (GO:0005912), *cell–cell adhesion* (GO:0098609), *cell adhesion* (GO:0007155), *focal adhesion* (bta04510), *cell adhesion molecules* (D015815), *gap junction* (bta04540), and *adherens junction* (bta04520). Functional terms associated with cellular proliferation include *microtubule associated protein* (IPR026074), *neuroepithelial cell differentiation* (GO:0060563), *establishment of epithelial cell polarity* (GO:0090162), *positive regulation of epithelial cell migration* (GO:0010634), *microtubule organizing center organization* (GO:0031023), and *blood vessel development* (GO:0001568). These biological processes are involved in many stages of reproduction including conceptus attachment to uterine epithelium, proliferation, and differentiation of trophoblast cells for development of placenta, and proliferation of endothelial cells to establish fetal-maternal circulation^46,47^.

Finally, membrane permeability was another term associated with many significant gene-sets implicated in pregnancy loss, including *potassium channel, voltage dependent, EAG (IPR003949), positive regulation of potassium ion transmembrane transport* (GO:1901381), *glucose transporter type 1* (DO51272*), regulation of glucose transmembrane transport* (GO:0034765), *regulation of ion transmembrane transport* (GO:0034765), *membrane potentials* (D008564), *potassium ion transmembrane transport* (GO:0071805). Membrane permeability plays a crucial role in signal transduction, intercellular communication for exchange of gas and nutrients, and controls embryogenesis and growth^48^. Voltage gated potassium channels are also expressed in uterine smooth muscle and play a significant role in modulating uterine contractility during pregnancy^49^.

Overall, all these enriched gene-sets play crucial roles in pregnancy maintenance, placental and fetal development, via cell attachment, proliferation, changes in membrane permeability, immunological modulation at the fetal-maternal interface, and steroid metabolism. Dysregulation of biological and molecular processes associated with these gene-sets might impair placenta function and fetal growth, causing pregnancy loss.

### Gene signaling pathways

**Figure 3:**
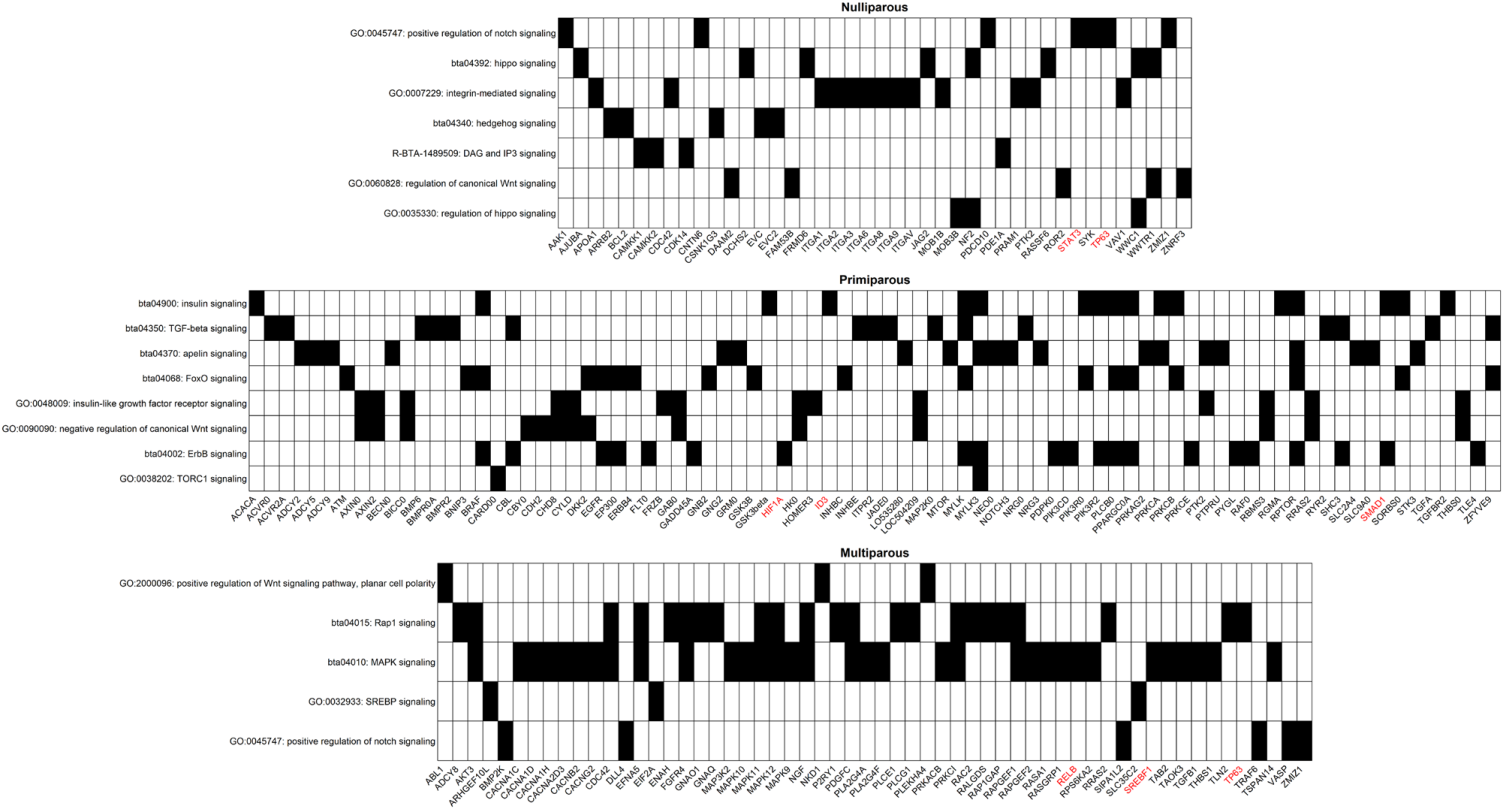
Heatmap plot of gene signaling pathways associated with pregnancy loss across the three parities. The y-axis displays the names of gene signaling pathways, and x-axis displays the names of genes involved in the pathways. Genes highlighted in red are recognized as bovine transcription factors.

Pregnancy maintenance, placental, and fetal development are characterized by cellular processes such as cell proliferation, migration, differentiation, transformation, and apoptosis. These cellular processes are genetically controlled and depend on the activities of gene signaling pathways, which coordinate the cell activities leading to organogenesis^50^. Our enrichment analysis revealed a number of significant gene signaling pathways, including Integrin, DAG & IP3, Wnt, Notch, Hedgehog, Hippo, MAPK, Rap1, SREBP, TGFβ and mTOR, that are all important for placental and fetal development (Fig. 3). These developmental signaling pathways are involved in cellular functions needed for morphogenesis, tissues homeostasis and organogenesis. For instance, the Wnt pathway comprises a family of ligands that bind to multiple receptor complexes triggering several downstream signaling cascades, including Wnt/β-catenin dependent signaling pathways, non-canonical Wnt/planar cell polarity (PCP), and the Wnt/Ca^2+^ pathways^51^. The Wnt signaling pathways are involved in chorion–allantois fusion, placental angiogenesis, and trophoblast differentiation^52^. As a result, perturbations in the Wnt signaling pathways may lead to failure in chorioallantois fusion or defects in the formation of placental vessels, leading to intrauterine growth restrictions and pregnancy loss^53^.

Notch is another significant signaling pathway that mediates cell–cell communication. Notch signaling is involved in many organ formations, including vertebrate segmentation and skeleton development during fetal development^54^. Another important signaling pathway is Hedgehog signaling which is involved in trophoblast syncytialization, a critical process for placental development and maturation^55^. Additionally, Hedgehog signaling regulates fetal growth via IGF1R pathway in the placenta^56^. Moreover, Hippo signaling pathway is involved in angiogenesis for establishing feto-maternal circulation and regulating fetal growth. The Hippo pathway has essential roles in organ size control, tissues regeneration, cell fate decision and differentiation during fetal development^57^. The MAPK signaling pathway is involved in complex cellular processes like proliferation, differentiation, transformation, and apoptosis. Additionally, MAPK interacts with other intracellular signaling pathways such as steroid receptors for uterine cell proliferation^58^ and is involved in embryonic and yolk sac angiogenesis during feto-placental development^59^.

The Transforming growth factor β (TGFβ) is another significant signaling pathway implicated in cell growth and differentiation. The TGFβ signaling pathway regulates a variety of reproductive processes, including pregnancy, uterine growth, and fetal development^60^. The mTOR signaling pathway, another developmental signaling pathway, has critical roles in cell growth, survival, and metabolism in response to nutrients, growth factors, energy, and stress signals. The mTOR signaling pathway regulates placental growth, including oxygen and nutrient transport. Inhibition of placental mTOR signaling results in intra-uterine growth restrictions characterized by adverse perinatal outcomes such as neurodevelopmental dysfunction and fetal loss^61^. Furthermore, the Integrin signaling pathway is implicated in cellular differentiation and tissue assembly during embryogenesis and fetal growth as integrins mediate both signaling and adhesion^62^. Integrins connect extracellular matrix (ECM) components with the actin cytoskeleton and form ECM-integrin-cytoskeleton signaling axis which is important for proper development and function of fetal skeleton^63^. The DAG & IP3 is another important developmental signaling pathway that regulates cellular calcium signaling system which is involved in several cellular processes such as proliferation, growth, differentiation, and contraction of uterine smooth muscles during pregnancy^64^. The SREBP signaling pathway is involved in cholesterol and fatty acid biosynthesis, and cholesterol is required for fetal development^65^. The Rap1 signaling is implicated in several basic cellular functions, including cell–cell interactions, cell–matrix adhesion, proliferation, and regulation of cellular polarity during fetal development. The Rap1 signaling is also implicated in maintaining epithelial and endothelial cell junction integrity for maintaining the viability of fetus^66^.

Overall, all these significantly enriched gene signaling pathways play critical roles in pregnancy maintenance, placental, and fetal development. Interestingly, many of these gene signaling pathways are also involved in pregnancy establishment and early embryo development, suggesting that some key pathways are relevant throughout different stages of gestation.

## Conclusions

In this study, we performed an integrative genomic analysis to understand the genetic and biological basis of pregnancy loss in dairy cattle. Our study focused on pregnancy loss during the fetal period using data from cows that were first confirmed pregnant and subsequently diagnosed as non-pregnant in later pregnancy checks. Whole-genome scans identified at least seven genomic regions located on BTA2, BTA10, BTA14, BTA16, BTA21, BTA24, and BTA29 that explained more than 0.5% of the additive genetic variances for fetal loss. Interestingly, these genomic regions harbor candidate genes that have diverse biological roles in feto-placental growth, immune modulation, calcium signaling, vascularization, and organogenesis. Moreover, the enrichment analysis revealed at least seven relevant processes, namely cell–cell attachment, cellular proliferation, membrane permeability, immunity, calcium signaling, steroid metabolism and fetal development as significantly enriched with genes associated with pregnancy loss. Additionally, the enrichment analysis revealed a number of important gene signaling pathways, including Integrin, DAG & IP3, Wnt, Notch, Hedgehog, Hippo, MAPK, Rap1, SREBP, TGFβ, and mTOR. These signaling pathways have diverse roles in pregnancy maintenance, placental development, and fetal growth. Overall, this comprehensive study contributes to a better, deeper understanding of the genetic architecture of fetal loss in dairy cattle by unraveling genetic variants, individual genes and complex biological and physiological pathways responsible for pregnancy maintenance in dairy cattle. In addition, these findings can provide opportunities for improving pregnancy success in dairy cattle via marker-assisted selection.

## Supplementary Information

Supplementary Information 1.

Supplementary Information 2.

Supplementary Information 3.

Supplementary Information 4.

## Data Availability

The phenotypic and genotypic data analyzed in this study were obtained from North Florida Holsteins (Bell, FL), and Council on Dairy Cattle Breeding (Bowie, MD). These datasets were used under agreement, and hence, are not publicly available. However, data are available upon request to FP and with permission of North Florida Holsteins, and Cooperative Dairy DNA Repository.
