# Supplementary material for: Genes and pathways associated with pregnancy loss in dairy cattle
Source: Sci Rep. 2021 Jun 25;11:13329. doi: 10.1038/s41598-021-92525-0 (PMC8233422; doi:10.1038/s41598-021-92525-0)
Supplement: Supplementary file 1 — Supplementary Information 1. [file 41598_2021_92525_MOESM1_ESM.docx]

**Supplementary Information:**

**Table S1 List of significant gene-sets (Nulliparous)**

**Table S2 List of significant gene-sets (Primiparous)**

**Table S3 List of significant gene-sets (Multiparous)**
